# Development of the tonsillar microbiome in pigs from newborn through weaning

**DOI:** 10.1186/s12866-018-1176-x

**Published:** 2018-04-16

**Authors:** Luis Carlos Pena Cortes, Rhiannon M. LeVeque, Julie Funk, Terence L. Marsh, Martha H. Mulks

**Affiliations:** 1 Facultad de Ciencias Agrarias, Universidad de Pamplona, Pamplona, Colombia; 2 Comparative Medicine and Integrative Biology Program, College of Veterinary Medicine, Michigan State University, East Lansing, MI USA; 3 Department of Microbiology and Molecular Genetics, Michigan State University, East Lansing, MI USA; 4 Department of Large Animal Clinical Sciences, College of Veterinary Medicine, Michigan State University, East Lansing, MI USA

**Keywords:** Tonsil, Tonsillar microbiome, Microbiome development, Pigs, Weaning

## Abstract

**Background:**

Porcine tonsils are lympho-epithelial tissues, colonized by numerous bacteria and viruses, that act as a reservoir for both host-specific pathogens and zoonotic pathogens with a high potential of transmission to humans. There are no existing studies describing the development of the tonsillar microbiome. We sequenced 16S rRNA genes from tonsillar samples of pigs to follow the development of the microbial communities from birth through weaning. Samples derived from sows were also analyzed to determine potential sources for the tonsil microbiome in piglets.

**Results:**

The composition of the newborn piglet tonsil microbiome could be differentiated by litter and had strong similarity to the sow teat skin as well as sow vaginal microbiome. The tonsil microbiome in these young piglets was mainly dominated by members of the *Pasteurellaceae*, *Moraxellaceae*, and *Streptococcaceae* families, while there were some transient members of the microbiome that were abundant at specific times, such as *Staphylococcaceae* in newborns and *Fusobacteriaceae* and *Leptotrichiaceae* in weeks 2 and 3. The microbiome initially differed between litters but over the following 3 weeks the communities of different litters converged in composition and then diverged in week 4 due to a combination of changes and stresses associated with weaning, including a shift from milk to a solid diet, in-feed Carbadox^®^ and room change.

**Conclusions:**

A significant portion of the tonsil microbiome was acquired either at birth from the sow vaginal tract or within a few hours post-birth from the sow teat skin. Our data demonstrate a temporal succession in the development of the pig tonsillar microbiome through the first weeks of life, with a convergence in the composition of the microbiome in all piglets by 3 weeks of age. The combination of management practices associated with weaning coincided with dramatic shifts in the tonsillar microbiome.

**Electronic supplementary material:**

The online version of this article (10.1186/s12866-018-1176-x) contains supplementary material, which is available to authorized users.

## Background

Tonsils are lympho-epithelial tissues located at the junction of the oropharynx and nasopharynx that play a key role in surveillance of inhaled or ingested pathogens [1]. In pigs, the tonsils are colonized by numerous bacteria and viruses, and act as a reservoir for both host-specific pathogens and zoonotic pathogens with a high potential of transmission to humans [1–4]. Bacterial pathogens such as *Actinobacillus pleuropneumoniae, Streptococcus suis,* and *Salmonella enterica* are frequently found in tonsils of asymptomatic animals. Under conditions of stress, such as animal transport, these pathogens can spread to the lower respiratory and gastrointestinal tracts and be transmitted to other animals, including humans [5]. It has been suggested that the tonsillar microbiome plays a preventive role in host colonization by pathogenic microorganisms [6–8] and also exerts regulatory roles in maintaining immune homeostasis, providing resistance to infection [9, 10].

In contrast to the rising number of studies characterizing the intestinal microbiome of mammals, there are few available studies characterizing the tonsillar microbiome and its development in those species. We have previously characterized the core microbiome of the tonsils in healthy 18–20 week old pigs [11]. In that study, members of the families *Pasteurellaceae, Moraxellaceae, Streptococcaceae, Fusobacteriaceae*, *Veillonellaceae, Enterobacteriaceae, Neisseriaceae,* and *Peptostreptococcaceae,* as well as the order *Clostridiales,* constituted the core tonsil microbiome in these grower-finisher pigs. Tonsils remain an under-explored habitat of the mammalian microbiome, and how the tonsil microbiome is established, how the structure of this community affects acquisition and carriage of pathogens, and how it contributes to health and disease, in animals or in humans, are not well understood.

Currently, there are no studies following the development of tonsillar microbiomes in humans or in pigs, while other microbiomes, such as the intestinal microbiome in mammals, have been more broadly studied in the last decade. Studies conducted in pigs and humans have suggested that the development of the intestinal microbiome is a gradual and successional process [12–15] with a significant fluctuation after weaning [16]. The cessation of milk feeding as well as supplementation of other diets and feeding sources generate changes in the intestinal microbiota [17–21]. It is unknown if a similar successional process occurs in tonsils.

It has been shown for humans and pigs that in the development of intestinal microbial communities there is, initially, substantial individual variation in community composition that tends to shift over time, eventually becoming similar in the major phyla across individuals [14]. Environmental factors such as the maternal microbiota can play a relevant role in the initial development of the intestinal microbiota [12, 14, 22]. Nevertheless, the developing microbiome is composed of interacting bacteria and not simply randomly assembled microorganisms [13].

The goal of the current study was to utilize culture-independent, high-throughput sequencing of 16S rRNA genes to follow the development of the tonsillar microbial communities in pigs from birth through weaning.

## Methods

### Animals

The Michigan State University Institutional Animal Care and Use Committee approved this study and the animal procedures. Ten crossbred sows (Yorkshire x Hampshire) from the high health status herd at the Michigan State University Swine Teaching and Research Center were used in this study, with full approval from the facility coordinators and the Department of Animal Sciences. This herd has no history of recent respiratory disease and is considered free of *Actinobacillus pleuropneumoniae*, *Mycoplasma hyopneumoniae*, and Porcine Respiratory and Reproductive Syndrome virus by medical history. This herd experienced a recent outbreak of porcine epidemic diarrhea virus (PEDV), which was under control at the time this study was initiated. The herd has a history of vaccination against porcine circovirus type 2 (PCV2), erysipelas and atrophic rhinitis.

This herd was managed as a farrow to finishing facility with ~ 200 sows. Newborn piglets received a single intramuscular injection of Iron-Dextran. Piglets were weaned at 3 weeks of age (21 to 24 days – average weight 18 pounds) and moved to a nursery room, with litters maintained as pen mates. The weaned piglets were introduced to a solid diet based on a pellet ration (Pig 1300^®^, Akey Nutrition, Brookville, OH) supplemented with Carbadox^®^ at a dose of 50 g/ton.

Individually identified sows of different parity, from primiparous (pregnant for the first time) to multiparous (pregnant multiple times), were purposely selected for this study and included two first parity sows (ear tags 1700 and 1707, respectively), 1 s parity sow (1631), one fifth parity sow (1445), one sixth parity sow (1402) and one tenth parity sow (1711). Four piglets from each of these six sows, selected randomly, were sampled within a period no longer than 8 h post-birth (PB) (PB were piglets which might have interacted with other piglets or the sow before sampling) and then at 1, 2, 3 and 4 weeks of age. An additional four freshly delivered piglets were randomly selected (*N* = 16 piglets) from each of 4 crossbred Yorkshire x Hampshire sows (1 s parity sow (1785), two third parity sows (1604, 1760) and one fourth parity sow (1704)), and sampled immediately at birth (AB) (AB piglets were sampled before they had any contact with external sources other than vaginal sources), avoiding any contact of the piglet with either the sow teats or the pen environment prior to sampling, with the purpose of determining the status of the microbiome right at birth. The litter from sow 1604 was sampled in 2014; the remaining three litters were sampled in 2015.

### Collection of microbiome samples

Samples of the tonsil microbiome were collected from sows and piglets using either cytology brushes (Cytosoft™, Medical Packaging Corporation, Camarillo, CA) for very small piglets or tonsil brushes developed by our group and validated in previous studies [11]. Collection of samples was as previously described [11]. Briefly, samples were collected at approximately the same time of day for each sampling time. Sow tonsillar samples were collected before they were fed. The pigs’ movement was restricted either by holding them firmly wrapped with a towel, or by using a snare on larger animals. The mouth was held open by using a mouth speculum while the tonsils were brushed. Right and left tonsils were brushed for approximately ten times each, rotating the brush in a clockwise fashion. Brushes were removed from the pig’s mouth and placed into a 50 ml sterile test tube containing 20 ml of 80% ice-cold ethanol. Samples were stored at − 20 °C until processed.

The sow vaginal microbiome was sampled by introducing a sterile cotton swab approximately 8 cm into the vaginal tract and rubbing the vaginal walls with the swab while turning the swab in a clockwise fashion. The teat microbiome was also collected by using a cotton swab and rubbing the teat surface of at least 10 teats per sow. Vaginal and teat swabs were placed individually into 50 ml sterile test tubes containing 20 ml of 80% ice-cold ethanol. Samples were stored at − 20 **°**C until processed.

The sow fecal microbiome was sampled by collecting approximately 5 g of feces directly from the rectum. Samples were placed individually into 50 ml sterile test tubes containing 20 ml of 80% ice-cold ethanol. Samples were stored at − 20 **°**C until processed.

### Isolation of community DNA

Sample extraction was performed as previously described [11]. Briefly, the 20 ml of 80% ice-cold ethanol containing the brushes, swabs or feces with the microbiome samples were thoroughly vortexed for 1 min, divided into equal volumes and transferred to two sterile acid washed Corex**®** tubes, and centrifuged in a refrigerated Sorvall SS-34 rotor at 16,000 x g for 30 min. After centrifugation, the supernatant was removed and discarded. The pellet of one tube was suspended in 5 ml of ice-cold 80% ethanol and archived at − 20 **°**C. The second pellet was suspended in 0.25 ml of phosphate buffered saline, pH 7, and transferred to PowerBead tubes (MoBio Laboratories, Carlsbad, CA) and vigorously shaken for approximately 2 min at room temperature using a MiniBeadBeater-16 (BioSpec Products, Inc., Bartlesville, OK). An exception to this protocol was followed with fecal samples, where both pellets were suspended in 0.5 ml of phosphate buffered saline, pH 7. Community DNA was then extracted using a PowerSoil DNA Isolation Kit (MoBio Laboratories, Carlsbad, CA) following the manufacturer’s instructions. The concentration of extracted community DNA was determined by spectrophotometry, using a Nanodrop (Thermo Scientific, Wilmington, DE). Each sample was then split in two vials; one was archived at − 80 **°**C and the other was processed for sequencing.

### Illumina sequencing and sequence analysis

For Illumina sequencing, samples were processed at the Michigan State University Research Technology Support Facility (RTSF) using an Illumina MiSeq platform. Negative controls consisting of either DNA-free water or MoBio C6 solution (“blank library controls”, [23]) and positive controls consisting of either *Escherichia coli* DH5α genomic DNA or a well-characterized activated sludge polymicrobial community [24] were included in the sequencing runs. Briefly, the V4 region of the 16S rRNA gene of the community DNA was amplified using uniquely indexed primers for each sample, as described by Caporaso [25]. After PCR, amplification products were normalized using an Invitrogen SequalPrep normalization plate. The normalized samples were pooled and PCR reaction cleanup was done with AMPure XP beads. After quality control and quantitation, the pool was loaded on an Illumina MiSeq v2 flow cell and sequenced with a 500-cycle v2 reagent kit (PE250 reads). Base calling was performed by Illumina Real Time Analysis Software (RTA) v1.18.54 and output of RTA demultiplexed and converted to FastQ files with Illumina Bcl2fastq v1.8.4.

Amplicon analysis was performed using the open-source, platform-independent, community-supported software program mothur v.1.38.0 (http://www.mothur.org) [26]. Processing of the raw sequencing data was done according to the mothur standard operating procedure (http://www.mothur.org/wiki/MiSeq_SOP) [27]. Alignment was accomplished using the mothur-formatted version 123 of Silva 16S rRNA gene database [28]. Sequences were classified and any sequences classified as Chloroplast, Mitochondria, unknown, Archaea, or Eukaryota were removed from the data set. Subsampling at 5907 sequences per sample was performed, followed by a preclustering of the sequences and removal of chimeric sequences using a mothur-formatted version of the Ribosomal Database Project (RDP) training set version 14 and uchime, based on the mothur protocol. Sequences were classified into Operational Taxonomic Units (OTUs) of ≥97% sequence identity. Singleton and doubleton reads were removed, followed by subsampling at 3776 sequences per sample. Because the negative controls consistently showed high levels of contaminants that skewed the results seen with some of the low biomass samples such as those from the newborn piglets, especially those from Litter 1700, 4 OTUs (*Ralstonia*, *Bacillaceae 1*, *Burkholderia,* and *Brevundimonas*) were also removed from the data set prior to final analysis. A SIMPER comparison of Litter 1700 piglet and sow samples to the relevant negative controls showed 66.1% or greater dissimilarity between the negative controls and the pig samples, rejecting the hypothesis that the sequence data for Litter and sow 1700 samples is due to contamination only [23]. Therefore these samples were included in the final data set. For the final analysis of the data, samples were subsampled to 1979 reads per sample. The full data set analyzed is given in Additional file 1: Table S1.

### Diversity and statistical analysis

The statistical analysis was performed using a clustering cutoff of 3% for the processed sequences. Mothur output files were used to estimate diversity indexes and core microbiomes. PAST3 was used for generation of the UPGMA dendogram file, SIMPER, coordinate analysis of the samples, two dimensional scatter plot and 95% concentration ellipses [29]. ImageJ was used to measure the area of the ellipses for the two dimensional scatter plot [30]. Dendogram construction was performed using FigTree v.1.4.2. (http://tree.bio.ed.ac.uk/software/figtree/). RStudio (Version 0.99.446; https://www.rstudio.com/) and libraries: gplots (https://CRAN.R-project.org/package=gplots), plot3D (https://CRAN.R-project.org/package=plot3D), and rgl (https://CRAN.R-project.org/package=rgl) were used to generate heatmaps and tridimensional scatter plots, respectively. Inkscape 0.91 (https://inkscape.org/en/download/mac-os/), was used to process images and edit labels.

### Availability of supporting data

Raw sequence data is available at the NCBI database (SRA accession number: SRP110992) and the code for the mothur analysis is available at (https://figshare.com/s/2c98593a953cc9bb1366).

## Results

A total of 171 samples derived from the tonsillar microbiome of piglets and sow tonsils, teat skin, vagina and feces as well as control samples were processed. One hundred forty-four pig samples were collected in Spring 2014, and an additional 12 pig samples were collected in Fall 2015. A total of 26 samples with less than 3776 final reads were not included in the final analysis. One of the approaches that we used was comparative analysis of core microbiomes. We defined the core microbiome for a specific litter as the OTU members of the microbiome that were present in at least 75% of piglets of a litter, when litters had at least 4 samples; otherwise core OTUs were defined as OTUs present in all the samples. For the sow samples, where 6 samples were analyzed, we considered OTUs present in 66.6% of the samples to be core OTUs. Further, core OTUs were defined as present in a minimum relative abundance equal or higher than 1% and/or 0.1% of total reads for a selected litter and/or period (Additional file 2: Table S2).

### The tonsillar microbiome found in PB samples clusters by litter

Among the 156 samples collected, 40 samples contain the tonsillar microbiome of newborn piglets (Table 1). Twenty four of these 40 samples were collected from PB piglets and the remaining 16 samples were collected from AB piglets. Six samples were discarded from the analysis due to low number of reads.

**Table 1:** Identification of sows, litters, sample collection and times of collection

Sow ID	# sow parity	Litter members	# piglets	# analyzed	Newborns Sampling	Sow samples			
						Feces	Teats	Tonsils	Vaginal
1700	1	10,11,12,13	4	4	PB	1	1	1	1
1707	1	31,32,34,35	4	3	PB	0	1	1	1
1631	2	15,16,17,18	4	4	PB	0	1	1	1
1445	5	36,39,40,42	4	4	PB	0	1	1	1
1402	6	1,2,4,6	4	3	PB	0	1	1	1
1711	10	22,23,24,26	4	4	PB	1	1	1	1
1785	2	49,50,51,52	4	3	AB	0	0	0	0
1704	4	66,67,68,69	4	4	AB	0	0	0	0
1760	3	79,80,84,85	4	3	AB	0	0	0	0
1604	3	43,44,45,46	4	2	AB	0	0	0	0
Total			40	34		2	6	6	6

PB. Samples were collected from newborn piglets in a period not greater than 8 h post birth

AB. Samples were collected from newborn piglets immediately after birth

**Fig. 1:**
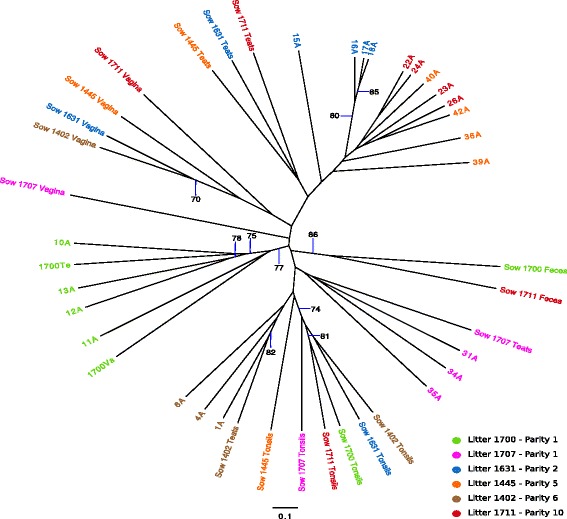
Unrooted Bray-Curtis dendrogram of PB and Sow microbiomes. The samples are color coded by the source. Bootstrap values higher than 70% at 1000 iterations are shown

A dendrogram clustering of PB piglet samples and sow derived samples, based on the Bray-Curtis dissimilarity index (Fig. 1) showed that samples from each litter clustered together with littermates. Microbiomes derived from piglets from primiparous sows (Litter 1700 and 1707) clustered in separated clades, as did the piglets from sow 1402. In general, PB piglet samples were more closely associated with samples derived from the sow teat microbiome as opposed to other sow samples. However, piglets from litter 1700 also clustered with the sample from the sow vaginal microbiome.

Samples from the sow tonsils and sow feces clustered together by source. Samples from the vaginal tract of multiparous sows clustered together, but were distinct from those from the two primiparous sows.

**Fig. 2:**
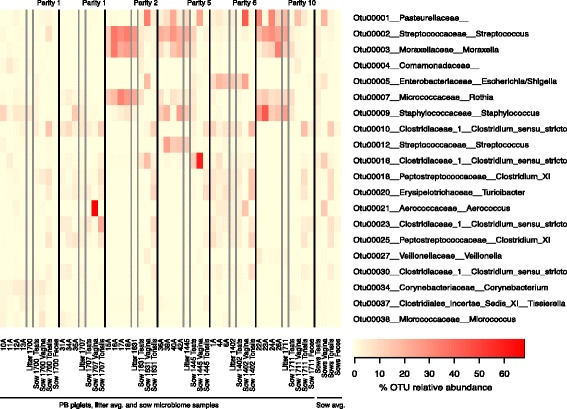
Thirty most abundant Operational Taxonomic Units (OTUs) for piglets and sow samples. Heat-map showing the relative abundance of the top 20 OTUs identified for all samples. The figure shows the relative abundance of the OTUs for each PB piglet and sow samples, as well as the average for the litters and sow samples

A heat-map representation of the major OTUs reveals the differences driving the clustering (Fig. 2). In the PB piglets from sows 1445, 1631, and 1711, all multiparous sows, the most abundant OTUs were *Pasteurellaceae, Streptococcus*, *Moraxella, Rothia,* and *Staphylococcus* (OTUs 001, 002, 003, 007, and 009, respectively). PB piglets from sow 1402 (parity 6) contained significant numbers of *Pasteurellaceae, Streptococcus*, and *Moraxella* (OTUs 001, 002, and 003) but also contained large numbers of *Enterobacteriaceae* (OTU005) and *Clostridium* sensu *strictu* (OTU010). In contrast, piglets derived from primiparous sows 1700 and 1707 had a very low abundance of *Pasteurellaceae*, *Streptococcus*, and *Moraxella* (OTUs 001, 002 and 003), but a high abundance of *Staphylococcus* (OTU009) as well as several anaerobic organisms.

### A significant proportion of the microbiome of the piglets came from maternal sources

We processed samples derived from teat skin, vagina and tonsil from six sows belonging to the PB group (Table 1), and fecal samples from two of these sows. We compared and traced which OTUs were identified as core microbiome for each litter and if they were identified as core for microbiomes derived from the different sow sources (Additional file 2: Table S2).

The OTUs identified as core for PB litters varied between litters. As was seen in Fig. 2, *Pasteurellaceae*, *Streptococcus* and *Moraxella* (OTUs 001, 002 and 003) were identified as core with a minimum relative abundance equal or higher than 1% and were found in high proportions in most piglets from multiparous sows but not piglets from primiparous sows. When sow sources for these three OTUs were examined, OTU001 *(Pasteurellaceae)* was identified as a core organism in the sow vaginal tract, representing on average 20.1% of the vaginal microbiome. However, OTU001 was present in only low amounts in the vaginal tracts from primiparous sows. In contrast, OTU002 (*Streptococcus)* was found in both vaginal and teat skin samples, while OTU003 (*Moraxella*) was found mainly in teat skin samples; both were more prevalent in samples from multiparous sows. In addition, *Rothia* (OTU007) and *Staphylococcus* (OTU009) were identified in high proportions and as core for most PB litters regardless of sow parity, as well as core for the sow teat microbiome, but not as part of the core microbiome for other sow samples.

These results suggested that the PB piglets had acquired new organisms from teat skin or other sources within the first few hours of life. To test this hypothesis, we collected samples from piglets immediately at birth (AB), prior to any contact with sources other than the uterus and the sow vaginal tract. These samples were very sparse in content, and unfortunately the resulting sequence data was dominated by organisms known to be frequent contaminants in DNA extraction kits and library preparation kits, including *Comamonadaceae*, *Sphingomonadaceae* and *Xanthomonadaceae* [23]. However, when these likely contaminants were deleted from the data, it was clear that the organisms most frequently seen were organisms associated mainly with the sow vaginal tract, including OTU002 *Streptococcus*, *Corynebacterium* (OTU034 and 051, and several anaerobic organisms including multiple OTUs of *Clostridiaceae and Peptostreptococcaceae.* OTU001 *Pasteurellaceae* was also found although in low numbers.

**Fig. 3:**
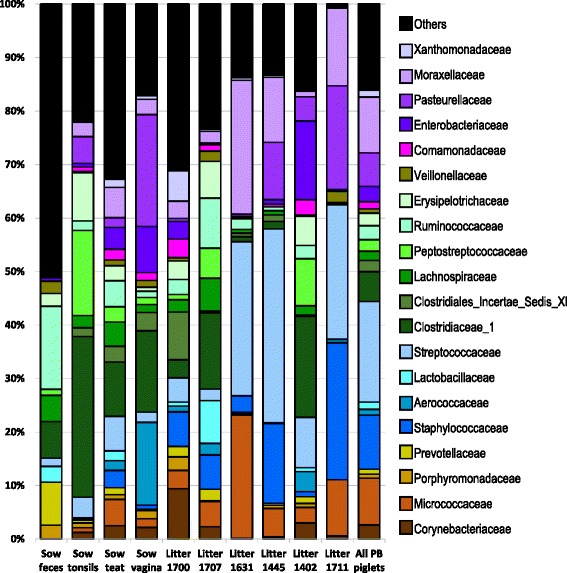
Twenty most abundant families identified in sows and PB microbiome samples. Bar plot shows the mean values for each family in Sow and PB samples, including each PB litter (percent of total OTUs)

An analysis of the microbiome derived from sow sources as well as from PB piglets at the family level showing the 20 most abundant taxa is presented in Fig. 3. These taxa generally include multiple OTUs. *Streptococcaceae* (20 OTUs), *Staphylococcaceae* (4 OTUs), *Micrococcaceae* (10 OTUs), *Pasteurellaceae* (21 OTUs) *Moraxellaceae* (29 OTUs) and families belonging to the *Clostridiales* (746 OTUs) were present in high abundance in the PB piglets, and were also found in the sow vagina samples and/or the sow teat skin samples.

### Tonsil communities of newborn piglets differed initially between litters but by 3 weeks of age clustered together reflecting similar composition

**Fig. 4:**
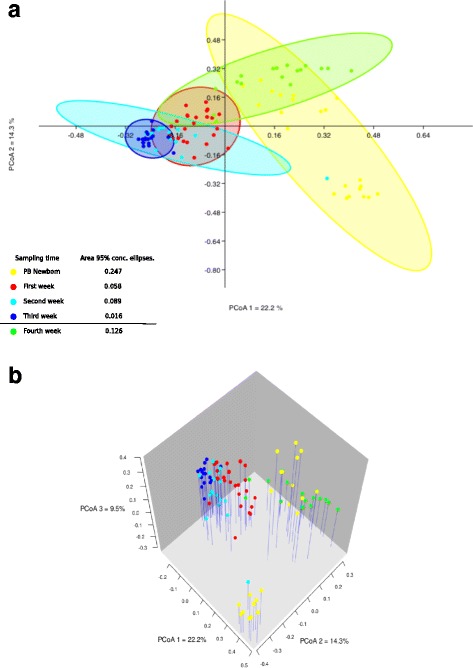
Principal Coordinate Analysis (PCoA) characterizing the tonsillar microbiome from PB piglets through the different sampling times. Two dimensional plotting illustrating the distribution of microbiome in the first two axis, the 95% concentration ellipses for newborn piglets through 4 weeks and the relative area for the ellipses (**a**). Three-dimensional plot illustrating the main three axes for the distribution of the microbiome of PB piglets since newborn through 4 weeks (**b**)

We inquired if, over time, the tonsillar communities of piglets reached a common microbiome. A principal coordinate analysis (PCoA) based on Bray-Curtis distances for the PB newborn through week 4 samples (Fig. 4a and b) showed a dynamic pattern in the samples. The PB newborns were widely spread although two distinct clusters were detectable. Over the next 3 weeks the microbiomes formed increasingly tighter clusters reflecting greater similarity as the animals aged. However, in the fourth week there was a dramatic shift, where, instead of continuing to cluster together more tightly as observed in previous weeks, samples were scattered.

Further analysis using a SIMPER approach based on Bray-Curtis distances (Additional file 3: Table S3) reinforced what we observed in the PCoA plots. Microbiome samples from PB piglets were dissimilar when compared with others and the value of dissimilarity fluctuated, with the lowest dissimilarity being 45.8% for litter 1445 vs 1711 and the highest being 94.04% for litter 1631 vs 1707. Although there was a substantial variability in the overall dissimilarity between litters, as time advanced the dissimilarity value between litters decreased and reached the lowest values in the third week, when values were as low as 23.5% for litters 1402 vs 1707 and the highest was 37.3% for litters 1402 vs 1445.

**Fig. 5:**
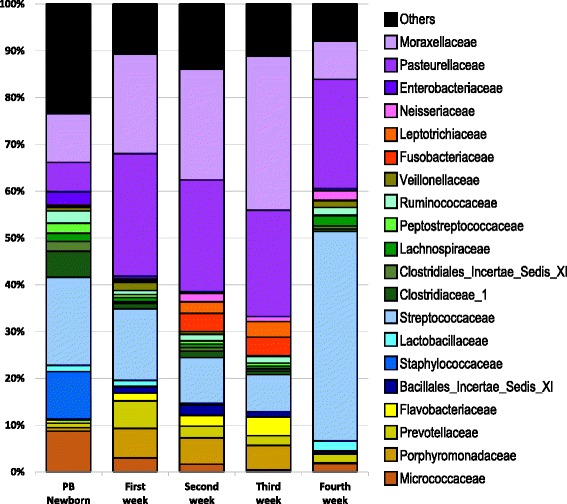
The abundance of the twenty most common families in PB piglets sampled from newborn through 4 weeks. Bar plot shows the mean values for the twenty most abundant families identified over the first 4 weeks of life (percent of total OTUs)

To visualize the temporal patterns of distribution of different bacterial families, we charted the mean value for the 20 most commonly identified families per sampling time (Fig. 5). While there were litter to litter variations in the microbiome of PB newborn piglets, as shown in Fig. 3, the differences between litters disappeared through the successional development of the microbiome in the following weeks.

Members of the families *Micrococcaceae* and *Staphylococcaceae* were abundant in PB newborns but decreased drastically and almost disappeared in the following weeks*.* By 1 week of age, the tonsil microbiome was dominated by members of the *Pasteurellaceae*, *Moraxellaceae*, and *Streptococcaceae* families, a dominance that remained throughout the lives of these pigs (unpublished data). In contrast, some members of the microbiome appeared and disappeared over time, such as *Porphyromonadaceae* and *Flavobacteriaceae* which appeared at weeks 1 to 3, as well as *Fusobacteriaceae* and *Leptotrichiaceae* that were present during weeks 2 and 3.

### The transition between third and fourth week represents a critical period for the development of the microbiome

According to MSU farm management practices, piglets were weaned between the third and fourth week. We investigated if the management practices experienced by the piglets (weaning, shift to a nursery room and introduction to a pelleted ration supplemented with Carbadox^®^), correlated with the timing of shifts in community structures.

An analysis of the members of core microbiome (Additional files 4 and 5: Table S4 and Table S5) demonstrated that for the third week, OTU001 (*Pasteurellaceae*), OTU002 (*Streptococcus*), OTU003 (*Moraxella*), OTU006 (*Porphyromonas*), OTU017 (*Fusobacterium*) and OTU026 (*Flavobacteriaceae*), identified as core with a minimum relative abundance equal or higher than 1%, were shared among all the litters, and represented on average 70.1% of the identified OTUs. In addition, OTU012 (*Streptococcu*s), OTU15 (*Prevotellaceae*), OTU018 (*Peptostreptococcaceae*) and OTU024 (*Bacillales Incertae Sedis XI*) were core members at a minimum relative abundance of 0.1% or higher. OTU001 (*Pasteurellaceae*), OTU002 (*Streptococcus*) and OTU003 (*Moraxella*) dominated the microbiome, together representing on average 59.5% of the identified OTUs. An analysis at the family level (Fig. 5) for the same period shows the families *Moraxellaceae* and *Pasteurellaceae* as the most abundant families with an abundance of almost 55.6%, followed by members of the families *Streptococcaceae*, *Porphyromonadaceae*, *Fusobacteriaceae*, *Leptotrichiaceae* and *Flavobacteriaceae*, together with an abundance of 24.5%.

The fourth week showed a different panorama, where only two OTUs, OTU001 (*Pasteurellaceae*) and OTU002 (*Streptococcus*), were identified as core at 1% or higher for all the litters. However, in contrast to the third week, these two OTUs represented on average 67.6% of the identified OTUs. In the fourth week, members of the *Streptococcaceae* family increased dramatically 5–6 fold compared to week 3, *Pasteurellaceae* remained at the same levels, and members of *Moraxellaceae* decreased 4 fold. Members of *Porphyromonadaceae*, *Flavobacteriaceae*, *Fusobacteriaceae*, and *Leptotrichiaceae* decreased to negligible numbers in week 4.

Furthermore, a SIMPER analysis (Additional file 3: Table S3) indicated that the dissimilarity in percentages between litters showed changes; instead of continuing the downward trend as observed in previous weeks, the dissimilarity values generally increased by 10–20%, with the lowest being 32.04% between litters 1631 and 1402 and the highest 47.98% between 1700 and 1445.

## Discussion

In this study, we followed the development of the tonsillar microbial communities in pigs from birth through weaning. We focused on identification of the source of bacteria found in the tonsils, the successional development, and the apparent effect of weaning on the tonsillar microbiome.

Analysis of the microbiome in the PB piglets showed that the most abundant organisms were *Streptococcus*, *Staphylococcus, Moraxella, Rothia,* and *Pasteurellaceae* (OTUs 002, 009, 003, 007, and 001 respectively). Based on comparative analysis of putative source microbiomes and piglet tonsil microbiomes, as well as cultivation studies, we concluded that while the *Pasteurellaceae* and *Streptococcus* were most likely acquired from the sow vaginal tract during the birth process, *Moraxella, Staphylococcus* and *Rothia* were likely acquired from the sow teat skin (or milk, which we did not sample) with a few hours after birth.

To determine what organisms were actually acquired during birth, we collected samples from piglets immediately after they were born (AB piglets), prior to contact with teat skin or other sources except the sow vaginal tract. As described in results, these samples were very sparse and the microbiome identified in them was heavily biased by likely contaminants from either the DNA extraction kit or library construction kit used. This has recently become a recognized problem in microbiome studies on low microbial biomass samples [23, 31, 32]. While we ran appropriate positive controls and zero template library construction controls, we did not run DNA extraction controls with the same kit used in this study. We have done this with a different lot number of the same kit, and used the information from that experiment as well as other published studies [23] to help analyze the data on the microbiome in AB piglets.

When we removed what we considered to be contaminants from the data set for AB piglets, the remaining organisms included OTU002 *Streptococcus* and OTU001 *Pasteurellaceae*, both of which colonized, multiplied, and persisted for the life of these pigs. It should be noted that we have cultured *Streptococcus suis* from at least half of these AB piglet samples, as well as from sow vaginal samples, providing additional support for the conclusion that this organism is acquired during the birth process. We also identified organisms such as OTU016 *Clostridium* sensu *strictu* and two OTUs (034 and 051) identified as *Corynebacterium* that were found in the sow vaginal tract and in AB and PB piglets, but not older piglets, suggesting that some of the organisms acquired during birth do not persist in the tonsils.

If these organisms failed to adhere to the tonsil epithelium, they would be washed out of the oropharynx, and other organisms acquired from the vaginal tract, such as *Streptococcus*, that do adhere to the tonsil epithelium would be able to colonize and multiply. Further, once the piglets had contact with other sow sources such as the teat skin or milk, new colonizers such as *Staphylococcus* could be acquired, leading to a sequential development of the tonsil microbiome starting within the first few hours of life. The clustering pattern observed in Fig. 1, where samples from three of the four multiparous litters clustered together as a group with sow teat skin samples and litters born from primiparous sows clustered with the cognate teat and vaginal or just teat samples, reinforce that teat and/or vaginal samples from sow are the initial source of the microbiome for PB newborns. Our findings are supported by the results of other studies, such as the study of Mandar and Mikelsaar [33], which characterized the initial colonization of the external ear canal in newborn humans and compared the microorganisms found with the vaginal flora of their mothers and concluded that there is a significant influence of the vaginal microflora in the initial microbial population found in the newborns. Further, Bokulich et al. [12], studying the effect of antibiotics, birthmode and diet in the development of fecal microbiota in children during early life, suggested that early colonizers are transmitted to children from maternal microbiota. They found that children delivered vaginally shared more OTUs derived from the vagina than children delivered by ceasarean. Additionally, they identified that the infant fecal microbiota was initially associated with vaginal and rectal maternal microbiota, but later was more associated with maternal fecal microbiota. Further, a study of the development of the human intestinal microbiome suggested that the bacterial population detected in human infants in early stages of life might be determined by specific bacteria to which infant was previously exposed, based on similarity patterns observed between infant samples and maternal sources as breast milk and vaginal swabs [14]. These authors identified *Streptococcus* and *Staphylococcus* as well as other aerobes as first colonizers [14]. Similarly, we observed that piglets sampled within short period after being born had high proportions of *Streptococcus*, *Moraxella* and *Staphylococcus* in their tonsils.

Our results demonstrated a strong litter effect in the tonsillar microbiome in PB piglets (Fig. 3). However, over the following 3 weeks there was a gradual successional development in the tonsil microbiomes of all piglets and by the third week the microbiomes of all piglets from all litters were highly similar (Fig. 4). This was true even for a litter that did not share the same farrowing room, as was the case for piglets from litter 1445 (data not shown). This is in contrast to the reported development of the pig intestinal microbiome over the first few weeks of life, where no obvious effect of litter was seen [34].

In this successional development, some organisms such as *Staphylococcus* and *Micrococcaceae* that were found in high proportions in the PB piglets decreased dramatically within the next 2 weeks, suggesting a role only as initial weak colonizers. These organisms are commonly isolated from multiple skin locations [35, 36] and vagina [37] and are likely to be vertically transmitted from sow to offspring. Over the same period there was a concomitant increase in stronger colonizers, particularly members of the families *Pasteurellaceae* and *Moraxellaceae*, as well as maintenance of the levels of *Streptococcaceae*. These three families, which contain both commensals and pathogens that are residents of mucosal surfaces of animal and humans, comprise a large proportion of the tonsil microbiome throughout the lives of pigs. Members of the order *Clostridiales* were also identified as a small but consistent part of the microbiome throughout the first 3 weeks, which is not surprising since they were identified as part of the core tonsillar microbiome of pigs [11] as well as members of intestinal microbiome [15, 18, 34, 38].

Over this period we also found several transitory OTUs or families that appeared and disappeared at specific time points. For example, *Porphyromonadaceae, Prevotellaceae,* and *Flavobacteriaceae* appeared at week 1 and increased slightly over the next 2 weeks. Similarly*, Fusobacteriaceae* and *Leptotrichiaceae* appeared at weeks 2 and 3. By week three, this successional development of the tonsil microbiome of all of the piglets, regardless of litter, led to a distinct common consortium of bacterial species.

Similarly, Palmer et al. [14], studied the microbiome profiles from human infant stool samples and suggested that although initially the microbiome was very distinct between individuals, over time it converged towards a common profile. The authors followed the development of the intestinal microbiome in 14 human infants, and showed that there was a considerable variation in the colonization process among individuals. Each infant had a distinct arrangement of bacterial species that it acquired and maintained. The acquired microorganisms had a temporal pattern in which they appeared and disappeared; however, they reached a stable population over time, with some taxonomic groups persisting while the presence of other taxa was only transient [14]. The authors reported the occurrence of significant shifts in the population assembly which seemed to stabilize over time.

At 3 weeks of age, the piglets were weaned onto a solid ration containing the growth promoter Carbadox^®^ and were moved from the farrowing room to the nursery, while kept in groups with their littermates. Comparison of the microbiome composition for the third and fourth weeks showed a major shift associated with the significant stress event of weaning, with its environmental, social and feed changes. Our results are in concordance with other studies following the development of the intestinal microbiome in humans and pigs, which reported significant changes in microbiome composition associated with life events [13, 14]. In pigs, it has been demonstrated that the transition from nursery to weaning is associated with a significant change in the intestinal microbiota [18, 39]. However, the effect of this transition on the tonsillar microbiome has not previously been studied in pigs or humans. In this study, we have demonstrated that the transition from farrowing to a nursery room in parallel with weaning and supplementation of the diet with Carbadox^®^ coincided with a major shift in the tonsil microbiota.

The most obvious effect of weaning was the 5–6 fold increase in members of the *Streptococcaceae*, from an average of 8% before weaning to ~ 43% of the total identified families after weaning. These organisms were primarily members of the genus *Streptococcus*. There was a concomitant decrease for members of the *Moraxellaceae* family, which decreased approximately 4-fold, from ~ 32% before weaning to ~ 8% after weaning. In addition, *Fusobacteriaceae*, *Leptotrichiaceae*, *Porphyromonadaceae* and *Flavobacteriaceae* decreased dramatically. However, at the same time, the proportion represented by *Pasteurellaceae* remained constant.

It is not clear whether a single stress, such as change in food or change in environment or application of antibiotic, or a combination of stresses was responsible for the major disruption in the tonsil microbiome at this time. It has been demonstrated in multiple studies that the intestinal microbiota composition was deeply perturbed when the host was treated with antibiotics [8, 12, 14, 40–43]. In our study, the piglets were supplemented with Carbadox^®^ in food at the time of weaning. It was reported by Looft et al. [40] that the structure and composition of the intestinal community of pigs supplemented with Carbadox^®^ changed significantly, where the relative abundance of *Prevotella* increased associated with Carbadox^®^ administration as a result of decreased abundance of other bacteria. Another study correlating changes in microbiota with changes of diet during nursing and weaning found that the fecal population of *Prevotellaceae* increased ~ 50-fold in weaned pigs compared with nursing animals [18]. In our study, members of *Prevotellaceae*, a minor population in the tonsils, decreased slightly from 2.7% for week three to 1.9% for week four. We can only speculate about the opposite results identified in our studies, since we are comparing different niches (feces vs tonsils) and we did not have controls that were not fed Carbadox^®^ as this was not a goal of our study.

It has been suggested that the environment also plays a relevant role in the initial acquisition of the microbiome. Mulder et al. [10], followed the development of gut microbiota and the potential impacts of early environmental changes, and demonstrated a major impact in the microbial diversity related with those changes and that the impact of those changes are preserved through adulthood. One of the major changes experienced by the weaned piglets is the separation from the sow and the introduction to new food. A deep impact in the intestinal microbial composition has been seen associated with cessation of breast feeding and introduction to a different diet [19].

We observed members of the microbiota that were present all the time, some in high relative abundance while others were found in low abundance. Similarly, there were also transient members whose exact role in the development of the tonsil microbiome is unclear but worth investigating in future studies. It is possible that these transient organisms appeared as secondary colonizers but were displaced by other members of the microbiota, or that these microorganisms were adversely affected by the stressful event of weaning and/or the Carbadox^®^ supplementation and thus disappeared. We have an imprecise idea about the true role of Carbadox^®^ in the development of the tonsillar microbiome, since our study was not specifically intended to answer this question. We unfortunately did not collect sow milk samples, or samples from the pen/cage floor which might have given us a better idea of the possible sources of the PB and subsequent microbiome.

There are many questions that arise from our work that will be the subject of future research. However, this study lays the foundation of our knowledge of how the tonsillar microbiome develops in pigs in the first hours and weeks of age and how weaning affects this microbiome. To the best of our knowledge, this is the first published study that follows the development of the tonsillar microbiome in any mammal during the first weeks of life.

## Conclusions

Our data demonstrate a temporal succession in the development of the pig tonsillar microbiome through the first weeks of life. Many of the organisms found in the piglet oropharynx and tonsils immediately after birth appear to wash out rapidly, within the first few hours of life, while other organisms acquired from the vaginal tract, such as *Streptococcaceae* and *Pasteurellaceae*, colonize and multiply. Additional organisms are also acquired rapidly from the sow teat skin, and possibly from milk, and eventually also from feces. The composition of the PB newborn piglet tonsil microbiome initially can be differentiated by litter and clusters mainly with the sow teat skin microbiome. Nevertheless, over the next 3 weeks, the composition and structure of the tonsil microbiome reaches a common point of development, showing a high degree of similarity among all piglets, regardless of litter and just prior to weaning. However, there was a dramatic change in the post weaning tonsillar microbiome, which was likely engendered by a combination of change in diet, change in environment, and addition of in- feed antibiotic, demonstrating the effect that weaning management practices exert in shaping tonsillar microbial communities. This research demonstrates the need for further studies to elucidate the role of antibiotic supplementation of feed in the development of tonsillar microbial communities, specifically when administered during the highly susceptible time of weaning.

## Additional files

Additional file 1: **Table S1.** OTU table for the full dataset. (XLSX 1547 kb)

Additional file 2: **Table S2**. Core microbiome at OTU level for litters and sow samples. Excel sheet containing values for Core OTU identified for litters and sow samples at 1 and 0.1% of minimum relative abundance. (XLSX 20 kb)

Additional file 3: **Table S3.** SIMPER analysis between litters through different sampling times. Excel sheet containing values from SIMPER analysis between different litters through the sampling time (Birth through fourth week). (XLSX 10 kb)

Additional file 4: **Table S4.** Core microbiome at OTU level for third week**.** Excel sheet containing values for Core OTU identified for 3 weeks old piglets at 1 and 0.1% of minimum relative abundance. (XLSX 16 kb)

Additional file 5: **Table S5.** Core microbiome at OTU level for fourth week**.** Excel sheet containing values for Core OTU identified for 4 weeks old piglets at 1 and 0.1% of minimum relative abundance. (XLSX 15 kb)

## Abbreviations

AB: Neonatal piglets sampled at birth
NCBI: National Center for Biotechnology Information
OTU: Operational Taxonomic Unit
PB: Neonatal piglets sampled within 8 h post-birth
PCoA: Principal Coordinate Analysis
RDP: Ribosomal Database Project
SIMPER: Similarity percentages
UPGMA: Unweighted pair group method with arithmetic mean
